# Fungal exopolysaccharides as next-generation microbial biomaterials: molecular biosynthesis, structural architecture, and translational biomanufacturing strategies

**DOI:** 10.1186/s12934-026-03002-0

**Published:** 2026-05-25

**Authors:** Hassan A.H. Ibrahim, Yasser H. El-Halmouch, Dalia El Badan, Rana Diab, Sameh S. Ali

**Affiliations:** 1 National Institute of Oceanography and Fisheries, NIOF, Cairo, Egypt; 2 Botany and Microbiology Department, Faculty of Science, Kafrelsheikh University, Kafr El-Sheikh, 33511 Egypt; 3 Department of Botany and Microbiology, Faculty of Science, Alexandria University, Alexandria, Egypt; 4 Botany and Microbiology Department, Faculty of Science, Tanta University, Tanta, 31527 Egypt

**Keywords:** Microbial polysaccharide biosynthesis, Structure–function relationships, Metabolic and synthetic biology, Bioprocess optimization and scale-up, Sustainable biomanufacturing, Waste valorization

## Abstract

Fungal exopolysaccharides (EPSs) are increasingly recognized as structurally programmable microbial polymers with applications spanning biomedicine, materials engineering, food systems, and environmental technologies. While previous reviews have often addressed fungal EPS diversity, production variables, or application domains separately, an integrated framework linking biosynthesis, molecular architecture, process control, and translational manufacturing remains underdeveloped. This review positions fungal EPSs as next-generation biomaterials by integrating (i) biochemical and genetic regulation of EPS biosynthesis, (ii) structure–function mapping across major polymer classes, (iii) cultivation and downstream processing workflows that enable reproducible product specifications, and (iv) industrial translation pathways within scalable and sustainability-aligned biomanufacturing systems. Gene-cluster–resolved case studies and process-to-product design principles illustrate how metabolic flux, fermentation parameters, and polymer modification shape functional performance. Current bottlenecks—including strain-dependent variability, purification complexity, quality harmonization, and techno-economic constraints—are critically evaluated to distinguish laboratory potential from scalable feasibility. By shifting from descriptive cataloging toward platform-based engineering logic, this review provides a translational roadmap for rational fungal EPS design within standardized and application-driven manufacturing frameworks.

## Introduction

Exopolysaccharides (EPSs) are high-molecular-weight extracellular polymers synthesized by microorganisms as adaptive matrices that support survival, surface adhesion, and environmental resilience across diverse ecological niches [1, 2]. In fungi, EPS secretion is tightly coupled to mycelial development, surface colonization, and stress adaptation, contributing to structured community formation and ecological persistence. Depending on their spatial association, fungal EPSs may remain closely bound to the cell surface as capsular EPSs or be released into the surrounding environment as slime EPSs. When secreted polysaccharides assemble together with proteins, extracellular DNA, and other macromolecules, they form a hydrated extracellular matrix that provides mechanical stability and protection [3]. In surface-associated fungal communities, this matrix constitutes the biofilm extracellular matrix, within which EPSs represent the dominant structural polymeric component; in this context, these matrix-associated polysaccharides are referred to as biofilm EPSs [4]. Beyond these ecological functions, fungal EPSs have attracted increasing attention as microbial-derived biomaterials owing to their extracellular biosynthesis, intrinsic biocompatibility, biodegradability, and structural tunability [5]. 

The functional performance of fungal EPSs arises from pronounced chemical and architectural diversity. These polymers include both homo- and heteropolysaccharides with distinct monosaccharide compositions, glycosidic linkages, and branching patterns that collectively determine solubility, rheological behavior, conformational stability, and biological activity [4, 6]. EPS biosynthesis is governed by coordinated carbon flux distribution and metabolic control mechanisms responsive to nutrient availability, pH, aeration, temperature, and cultivation time, resulting in variability in yield, molecular weight, and structural composition [1, 7]. Representative EPS-producing fungi from Ascomycota and Basidiomycota generate polymers such as pullulan, schizophyllan, scleroglucan, and lentinan, which have established industrial and biomedical relevance [8, 9]. Advances in fermentation engineering and bioreactor optimization have improved production consistency, facilitating the transition of fungal EPSs from laboratory-scale studies toward scalable microbial cell-factory systems.

Extensive research has documented the biomedical potential of fungal EPSs, including immunomodulatory, antioxidant, antiviral, antibacterial, and antitumor activities [2, 10]. Defined structural motifs within EPS backbones interact with host cellular pathways to influence cytokine expression, immune-cell activation, and oxidative stress responses. These properties have enabled the development of EPS-based drug delivery matrices, vaccine adjuvants, wound-healing materials, and tissue-engineering scaffolds. Industrially, fungal EPSs function as rheology modifiers, flocculants, gel-forming agents, and film-forming polymers, supporting applications in food processing, cosmetic formulations, wastewater treatment, and enhanced oil recovery [11–13]. Furthermore, the capacity of fungi to metabolize agricultural residues and lignocellulosic wastes aligns EPS production with circular bioeconomy principles that prioritize resource efficiency, waste valorization, and reduced reliance on fossil-based polymers [14, 15].

Despite these advances, large-scale deployment remains constrained by strain-dependent productivity, downstream purification complexity, variability in molecular characteristics, incomplete regulatory harmonization, and limited integration of gene-level biosynthetic understanding with process-scale design [4, 16]. Although numerous reviews have addressed fungal EPS diversity, production variables, or application domains, biosynthesis, structural determinants, industrial processing, and sustainability considerations are often treated as separate themes. Consequently, the translational pathway from discovery to reproducible, scalable, and regulatory-aligned manufacturing remains fragmented. A cohesive framework that connects biochemical and genetic regulation to polymer architecture, links fermentation parameters to functional performance, integrates downstream standardization workflows, and situates EPS manufacturing within techno-economic and circular bioeconomy contexts is still needed [17].

The present review addresses this gap by positioning fungal EPSs as architecturally programmable biomaterials whose properties can be rationally engineered through coordinated control of biosynthesis, process design, and quality specification. Specifically, it integrates (i) biochemical and genetic regulation of EPS biosynthesis, including gene-cluster–resolved examples; (ii) structure–function mapping across major polymer classes; (iii) cultivation and downstream workflows that enable reproducible product specifications and regulatory alignment; and (iv) industrial translation pathways embedded within scalable and sustainability-aligned biomanufacturing frameworks. By shifting from descriptive cataloging toward platform-based engineering logic, this review provides a translational roadmap that connects molecular biosynthesis to standardized manufacturing systems, ultimately accelerating deployment of fungal EPSs as application-specific microbial biomaterials.

## Ecological diversity of EPS-producing fungi as a driver of polysaccharide innovation

Although EPSs have historically been associated primarily with bacteria and cyanobacteria, extensive research has demonstrated that their production is widespread across multiple microbial kingdoms, including fungi, yeasts, and microalgae [8, 18]. EPS-producing microorganisms inhabit an exceptionally broad ecological range, which reflects the adaptive advantages conferred by extracellular polymer secretion. In aquatic environments, including freshwater and marine ecosystems, EPSs form hydrated protective matrices that support microbial attachment, nutrient retention, and defense against osmotic stress, thereby promoting colonization of submerged surfaces and participation in biofilm communities. Microbial EPS production is also prominent in terrestrial habitats such as agricultural soils, where polysaccharides enhance soil aggregation, water retention, and interactions with plant roots, promoting survival under fluctuating moisture and nutrient conditions [19].

The ability to synthesize EPSs is particularly advantageous in extreme ecological niches. Microorganisms isolated from hot springs, deep-sea sediments, hypersaline lakes, and polar regions demonstrate significant EPS secretion as a biochemical strategy to withstand thermal shock, salinity stress, nutrient scarcity, and freezing conditions [20]. Such extremophilic strains frequently produce EPSs enriched in uronic acids or sulfated residues that improve metal-binding capacity, cryoprotection, and viscosity regulation, supporting their ecological fitness and offering unique industrial prospects.

Marine fungi constitute one of the most explored sources of novel EPS structures due to the chemical complexity of marine ecosystems. EPS-producing taxa such as *Aspergillus versicolor* [21], *Fusarium oxysporum* [22], *Botryosphaeria rhodina* MMGR [23], *Penicillium griseofulvum* [21], *Hansfordia sinuosae* [24], and marine yeasts including *Candida famata* [25] and *Cryptococcus flavus* [26] have yielded EPSs with distinctive biological activities and physicochemical behaviors that are not commonly observed in terrestrial species. The metabolic versatility observed among these isolates demonstrates that marine environments remain an underexploited reservoir for discovering unique EPS-producing strains with high commercial potential. Overall, the widespread environmental distribution of EPS-producing microorganisms, from nutrient-rich aquatic systems to energy-limited extreme habitats, demonstrates that EPS biosynthesis represents a conserved and evolutionarily significant mechanism. This ecological versatility broadens industrial opportunities by providing diverse microbial sources capable of generating structurally novel EPSs with desirable biotechnological, environmental, and medical functionalities.

## Structural and functional classification of fungal EPSs

**Fig. 1:**
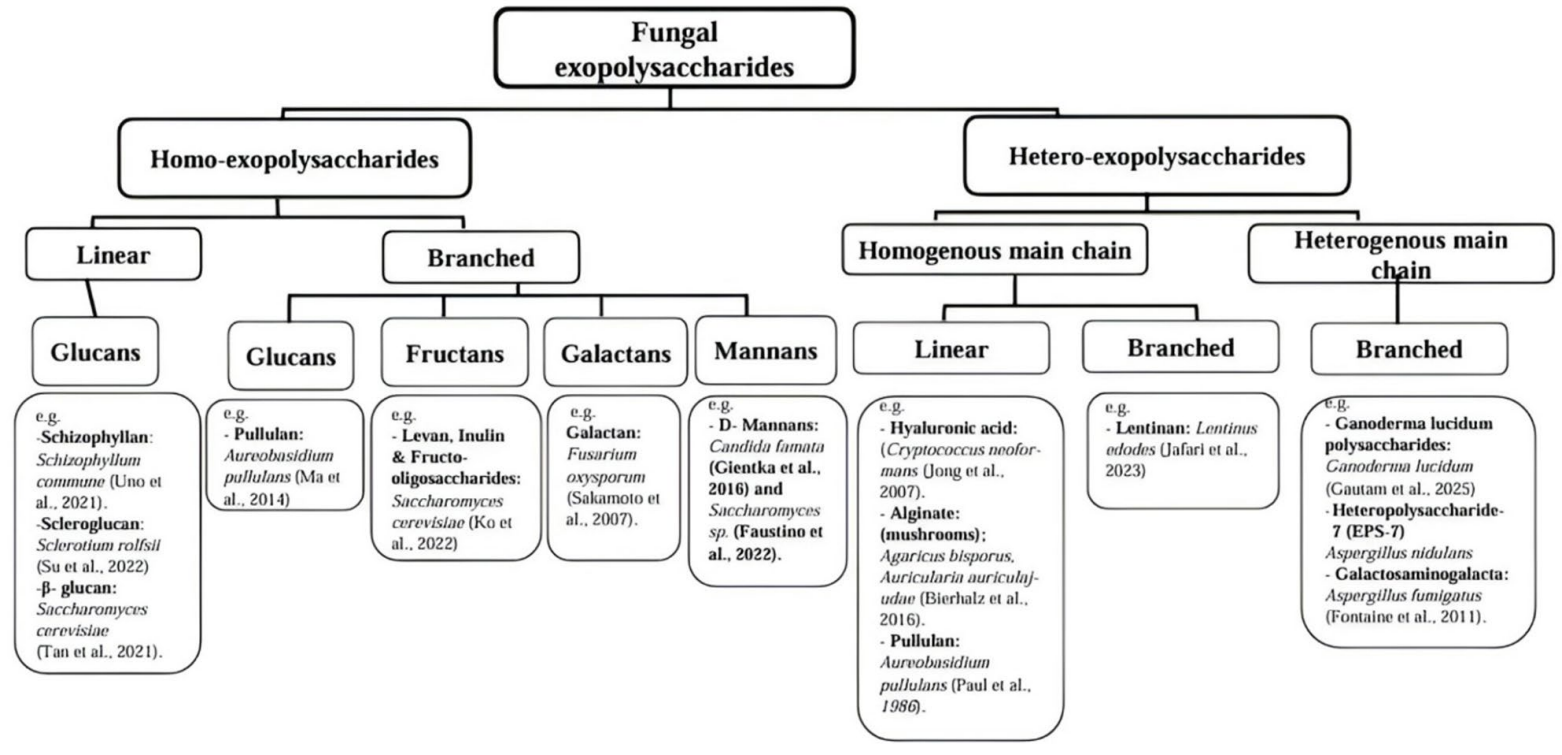
Schematic overview of fungal EPSs categorized into homo- and hetero-EPSs based on monosaccharide composition, main-chain uniformity, and branching architecture

Classification of fungal EPSs is most informative when it is treated not as a taxonomic exercise, but as a structure–function mapping system. Polymer architecture determines physicochemical behavior, and physicochemical behavior constrains biological and industrial performance. Accordingly, fungal EPSs can be organized along three interrelated structural axes: monosaccharide composition, chain topology, and linkage configuration [20, 27]. Figure 1 synthesizes this hierarchical framework. At the most fundamental level, EPSs are categorized as homo-EPSs or hetero-EPSs based on monosaccharide composition. Homo-EPSs consist of a single repeating sugar unit, while hetero-EPSs incorporate two or more monosaccharides arranged in defined sequences. This distinction influences not only chemical uniformity but also hydration behavior, charge distribution, and receptor interaction potential.

Within homo-EPSs, chain topology further differentiates linear and branched structures. Linear β-glucans and scleroglucans, produced by species such as *Schizophyllum commune* and *Sclerotium rolfsii*, exhibit extended conformations and strong intermolecular associations that support high viscosity and rheological stability. In contrast, branched homopolymers such as pullulan from *Aureobasidium pullulans* and fructans from yeasts including *Saccharomyces cerevisiae* display enhanced solubility and film-forming behavior due to altered chain packing and flexibility [6, 28]. Thus, branching modifies polymer mobility and intermolecular interactions, directly affecting gelation and barrier properties. Hetero-EPSs introduce an additional layer of architectural complexity through mixed sugar backbones. Linear heteropolysaccharides, including hyaluronic acid and alginate-like polymers from basidiomycetous fungi, exhibit strong hydration and viscoelastic characteristics attributable to distributed charge density and extended chain conformation. Branched heteropolymers such as lentinan from *Lentinus edodes* and polysaccharides from *Ganoderma* species demonstrate pronounced bioactivity, often linked to specific repeating motifs and branching frequencies that enable interaction with immune receptors [5, 13]. In these systems, compositional heterogeneity expands functional versatility by introducing chemically distinct interaction domains within the same macromolecule.

Beyond composition and topology, glycosidic linkage configuration is a decisive structural determinant. Linkages such as β-(1→3), β-(1→6), and α-(1→3) govern chain rigidity, helicity, and receptor recognition. For example, β-(1→3)-glucan backbones with β-(1→6) branching are frequently associated with immunomodulatory activity, as these motifs are recognized by innate immune receptors. Subtle shifts in linkage distribution can therefore alter conformational stability and biological specificity without changing overall monosaccharide composition. A final classification parameter concerns cellular localization. Capsular EPSs remain tightly associated with the fungal cell surface, whereas slime EPSs are released into the extracellular matrix [20]. This distinction influences extractability, diffusion behavior, and ecological function, and has practical implications for downstream processing strategies discussed later.

Taken together, the structural framework presented in Fig. 1 organizes fungal EPS diversity into a predictive matrix rather than a descriptive list. Monomer identity defines chemical potential, chain topology shapes rheological behavior, linkage configuration governs conformational and biological specificity, and localization affects functional deployment and recovery. This structure–function logic provides the necessary foundation for subsequent sections addressing taxonomic specialization, biosynthetic regulation, and process-level control of polymer design.

## Taxonomic specialization of fungal EPS biosynthesis

Taxonomic distribution of fungal EPS production is not merely descriptive; it reflects lineage-specific biosynthetic capacity shaped by evolutionary history. Among fungi, the phyla Ascomycota and Basidiomycota dominate EPS biosynthesis across both natural and engineered systems due to their expansive metabolic repertoires and adaptive ecological strategies (Osinska-Jaroszuk et al., 2015). Phylogenetic position influences glycosyltransferase diversity, sugar-nucleotide metabolism, secretion pathways, and ultimately polymer architecture. Basidiomycetes are frequently associated with structurally complex and highly branched polysaccharides, including β-glucan–rich polymers with defined immunologically active motifs. In contrast, many ascomycetes produce polymers with comparatively simpler architectures but strong rheological performance and fermentation robustness [8]. These lineage-dependent tendencies suggest that phylogeny constrains both backbone configuration and functional modification patterns, thereby shaping downstream material properties.

EPS localization further reflects taxonomic and physiological specialization. Polymers may remain closely associated with the cell surface as capsular EPSs or be secreted into the extracellular matrix as slime EPSs. Slime EPSs often consist of high-molecular-weight polysaccharides integrated with proteins and extracellular DNA, forming hydrated biofilm matrices that support nutrient adsorption, stress tolerance, and surface colonization. However, classification based solely on biochemical extraction origin can be confounded by contamination from cell-wall components or lysis products [3], underscoring the need for careful analytical discrimination.

From a translational perspective, both Ascomycota and Basidiomycota include species that have achieved industrial relevance. Within Ascomycota, EPS producers such as *Aspergillus versicolor* [21], *Fusarium oxysporum* JN604549 [29], *Penicillium griseofulvum* [21], and *Cordyceps sinensis* Cs-HK1 [30] illustrate fermentation-accessible systems capable of generating commercially viable polymers. In Basidiomycota, species including *Ganoderma lucidum* UF20706 [31], *Phellinus* sp. P0988 [28], and *Trametes versicolor* [32] produce bioactive polysaccharides with established therapeutic applications. The commercial success of pullulan, schizophyllan, scleroglucan, botryosphaeran, lentinan, grifolan, and lasiodiplodan, summarized in Table 1, reflects how lineage-specific biosynthetic traits can translate into scalable polymer platforms.

**Table 1:** Structural classification and validated producers of major fungal exopolysaccharides

Fungal EPS	Polymer class	Core structural motif	Validated producing species	Primary functional domain	References
Pullulan	Linear α-glucan homopolysaccharide	Maltotriose repeating units linked via α-(1→6)	*Aureobasidium pullulans*	Film-forming biomaterial; oxygen barrier	Ganie et al., [33]; Sugumaran and Ponnusami [34]
Schizophyllan	Branched β-glucan	β-(1→3) backbone with β-(1→6) branching	*Schizophyllum commune*	Immunomodulatory β-glucan	Kumar et al., [35]
Scleroglucan	Branched β-glucan	β-(1→3) backbone with regular β-(1→6) side chains	*Sclerotium rolfsii*; *Sclerotium glucanicum*	Rheology modifier; salinity-stable polymer	Survase et al., [36]
Botryosphaeran	Branched β-glucan	β-(1→3,1→6)-linked glucan	*Botryosphaeria rhodina*	Bioactive derivative platform	Geraldelli et al., [37]
Lentinan	Branched β-glucan	β-(1→3) backbone with β-(1→6) side chains	*Lentinula edodes*	Clinical adjunct immunomodulator	Sobieralski et al., [38]
Grifolan	Branched β-glucan	β-(1→3,1→6)-linked glucan	*Grifola frondosa*	Immunopharmacological research	Seo et al., [39]
Lasiodiplodan	Linear β-(1→6)-glucan	(1→6)-β-D-glucan	*Lasiodiplodia theobromae*	Antioxidant and functional derivatives	Ascensio et al., [40]

Yeasts, although central to industrial fermentation, remain comparatively underexplored for EPS development. Emerging work on extremotolerant and psychrophilic yeasts reveals promising biosynthetic potential beyond conventional strains. Members of Tremellomycetes, such as *Vishniacozyma victoriae* isolated from Antarctic environments, produce structurally distinctive EPSs associated with stress adaptation [41]. Aerobic cultivation of this strain yielded 4.5 g/L EPS after 120 h [42], while *Cryptococcus laurentii* AL65 produced approximately 3 g/L under low-energy operational conditions [43]. These findings indicate that non-conventional yeast clades may expand the accessible design space of fungal polysaccharides. Viewed through a framework lens, taxonomic specialization functions as a predictive layer in EPS discovery and engineering. Phylogeny influences enzyme repertoires, branching patterns, and secretion dynamics, thereby constraining polymer identity and functional capacity. Comparative genomics and metabolomics across fungal lineages therefore represent strategic tools for identifying novel biosynthetic clusters and expanding the structural landscape available for rational design. This taxonomic perspective bridges ecological diversity and molecular biosynthesis, setting the stage for mechanistic analysis of regulatory and enzymatic control systems in the following section.

## Biochemical and genetic regulation of fungal EPS biosynthesis

Fungal EPSs are synthesized through coordinated biochemical and genetic processes that integrate nutrient sensing, central carbon metabolism, polymer assembly, and extracellular export. These regulatory layers enable modulation of polymer architecture in response to environmental and physiological conditions. Elucidating EPS biosynthesis at the molecular level provides a framework for metabolic engineering strategies aimed at improving yield and controlling structural attributes relevant to industrial and biomedical applications. The biosynthetic system can be considered at two interconnected levels: (i) core metabolic pathways that direct carbon flux toward activated sugar precursors, and (ii) gene-cluster–encoded machinery that governs species-specific polymer assembly and modification.

EPS biosynthesis begins with carbohydrate uptake, typically glucose, via transporter systems responsive to nutrient availability. Intracellular glucose is phosphorylated to glucose-6-phosphate and subsequently converted to glucose-1-phosphate, a metabolic branch point that determines carbon allocation between energy metabolism and polysaccharide synthesis [44]. UDP-glucose pyrophosphorylase catalyzes formation of UDP-glucose, a principal activated sugar donor for glycan assembly. Regulation of sugar-nucleotide pools influences precursor availability and contributes to variability in polymer yield and composition.

Polymer elongation is mediated by glycosyltransferases that assemble sugar residues into defined backbones, often linked to lipid carriers during membrane-associated synthesis. Enzyme specificity determines linkage configuration, branching frequency, and repeating-unit organization, structural features that influence rheological behavior and biological interactions [45, 46]. Post-polymerization modifications such as acetylation, sulfation, or methylation alter charge distribution and interaction capacity. Export of completed polymers occurs through flippases and ABC transporters, after which polysaccharides may remain cell-associated as capsular EPSs or be released as slime EPSs into the extracellular matrix [44]. Differences in flux regulation, enzyme specificity, and post-synthetic modification contribute to the structural heterogeneity observed among fungal EPSs. Beyond central metabolism, specialized gene clusters coordinate precursor activation, polymer assembly, remodeling, and export. Conserved biosynthetic frameworks—including Wzx/Wzy-dependent systems, ABC transporter–associated pathways, and synthase-dependent mechanisms—interface with sugar-nucleotide metabolism to regulate chain length and modification [47]. Stress-responsive transcriptional regulators further modulate EPS production, linking environmental signals such as nutrient limitation, osmotic stress, or host interaction to polymer biosynthesis.

**Fig. 2:**
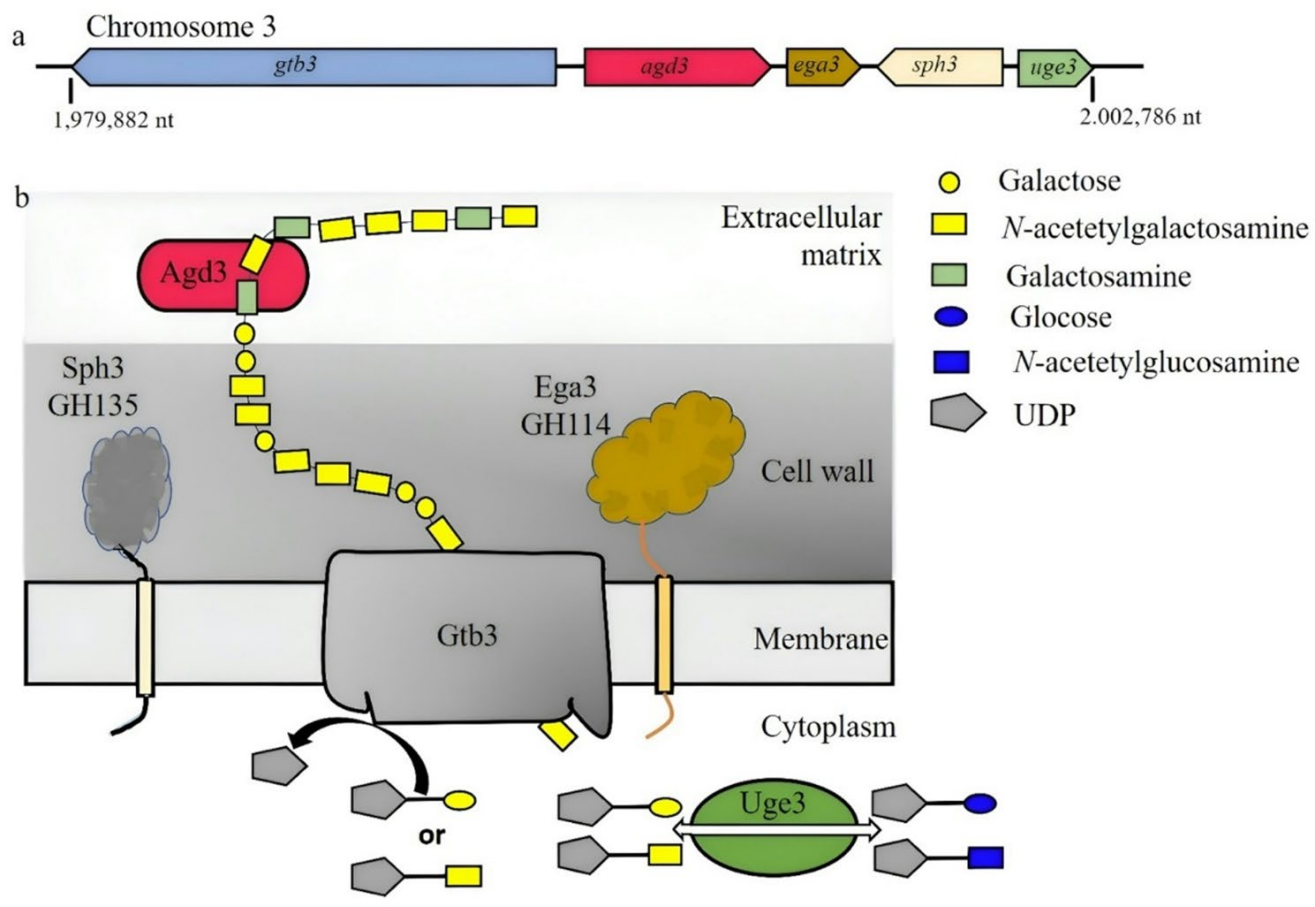
Genetic organization and biosynthetic model of galactosaminogalactan (GAG) production in *Aspergillus fumigatus*. **a** Genomic organization of the co-regulated GAG biosynthetic gene cluster located on chromosome 3. The cluster includes *gtb3*,* agd3*,* ega3*,* sph3*, and *uge3*, with gene positions indicated by nucleotide coordinates. **b** Schematic model of GAG biosynthesis, polymer processing, and extracellular assembly. The pathway illustrates the roles of Gtb3 in polymer elongation, Uge3 in precursor activation, Agd3 in partial deacetylation of the polymer, and Sph3 and Ega3 in extracellular remodeling and processing during matrix formation. The resulting polysaccharide is integrated into the extracellular matrix associated with the fungal cell wall. The depicted polymer architecture is representative and heterogeneous, based on bioinformatic analyses and experimental evidence. (adapted from [49])

A well-characterized example of gene-cluster–mediated control is the biosynthesis of galactosaminogalactan (GAG) in Aspergillus fumigatus. GAG consists of α-(1→4)-linked galactose (Gal) and N-acetylgalactosamine (GalNAc) residues and contributes to biofilm organization and host interaction [48]. The GAG biosynthetic cluster located on chromosome 3 (Fig. 2a) encodes enzymes responsible for precursor activation (Uge3), membrane-associated polymerization (Gtb3), extracellular remodeling (Sph3 and Ega3), and post-synthetic modification. The secreted deacetylase Agd3 introduces partial deacetylation, generating cationic regions required for matrix organization and virulence [49]. Deletion of agd3 impairs biofilm formation and attenuates pathogenicity (Fig. 2b), demonstrating how defined structural modification of an EPS directly influences biological function. Homologous clusters identified in other pathogenic fungi suggest that EPS-mediated host interaction is conserved across multiple lineages.

Characterization of these biosynthetic pathways enables targeted intervention at defined regulatory nodes. Manipulation of precursor flux, glycosyltransferase activity, or transcriptional control can modify polymer length, branching, and functional group distribution. Integration of pathway-level knowledge with synthetic biology tools and systems-scale metabolic modeling supports rational strain development aimed at improving production efficiency and structural consistency. In this context, fungal EPS biosynthesis can be approached as a genetically regulated platform linking metabolic control to material properties within scalable manufacturing systems.

## Biological and process determinants shaping fungal EPS yield and structure

Fungal EPS production is influenced by interactions among nutritional composition, cultivation parameters, and strain-specific regulatory networks. These variables affect not only overall productivity but also molecular weight distribution, branching architecture, and monosaccharide composition, thereby influencing rheological performance and bioactivity. Experimental studies demonstrate that cultivation conditions exert measurable control over polymer chemistry and viscosity [7]. Consequently, statistical optimization approaches—including response surface methodology, Plackett–Burman screening, Box–Behnken design, and central composite modeling—are routinely applied to define operational windows that maximize yield and structural consistency [20].

Medium composition is a primary determinant of EPS biosynthesis. Carbon source type and concentration influence metabolic flux toward sugar-nucleotide precursors and can modify polymer composition. Substrates such as glucose, sucrose, maltose, mannitol, and starch differentially affect carbohydrate content and chain characteristics. For example, starch-based media have been associated with increased carbohydrate fractions relative to glycerol fermentations [50]. Species-specific preferences are also reported: *Ganoderma lucidum*, *Inonotus levis*, and *Phellinus robustus* preferentially utilize glucose, whereas mannitol enhances EPS output in *Agaricus nevoi* and maltose supports higher productivity in *Cerrena maxima* and *Trametes versicolor* [9]. Mixed-carbon strategies can further increase yields; combining glucose and starch improved EPS accumulation in *Sclerotium rolfsii* more than fourfold compared with low-sucrose controls [51, 52]. In *Schizophyllum commune*, statistical screening identified a glucose–mannose mixture (40 g/L and 5 g/L, respectively) as optimal, yielding 8.16 g/L EPS under defined conditions [53].

Nitrogen availability modulates the transition from biomass formation to polysaccharide production. Organic nitrogen sources, including yeast extract and soy peptone, often support higher EPS titers than inorganic salts, likely due to provision of growth factors and amino acids [4]. Elevated C: N ratios generally favor polysaccharide accumulation during late exponential or stationary phases [54]. Micronutrients also influence polymer characteristics. Phosphate availability supports energy metabolism, while divalent cations such as Mg²⁺ enhance enzyme stability and precursor activation. Supplementation with Mg²⁺ and K⁺ increased EPS production in *Tuber sinense* by more than 130% relative to unsupplemented controls [55]. Additives including vitamins, fatty acids, and surfactants may further modulate secretion, although responses remain strain-dependent [56].

Environmental parameters exert additional control. Oxygen transfer efficiency affects both growth and polysaccharide synthesis in aerobic fungi. Increased aeration and agitation have been associated with higher EPS titers, as observed in *Aspergillus parasiticus* (0.41 g/L under agitation versus 0.18 g/L static) and *Aureobasidium pullulans* P56 (23 g/L at 1 vvm aeration) [57, 58]. Excess oxygen, however, may induce oxidative stress and suppress polysaccharide pathways [59]. Culture pH influences enzyme activity and polymer architecture; most species exhibit optimal production under mildly acidic conditions (pH 3.0–6.5), although broader tolerance ranges are reported [60, 61]. Temperature similarly shapes EPS yield and composition, with optimal production commonly observed between 22 and 30 °C [62]. Extended fermentation beyond stationary phase can result in partial depolymerization and reduced molecular weight fractions [4, 61].

Strain-level genetic and regulatory characteristics ultimately determine intrinsic productivity. Variability in glycosyltransferase repertoires, precursor mobilization, and export systems can produce substantial yield differences within the same species [54]. Environmental stresses, including osmotic or thermal shifts, may act as regulatory signals that enhance EPS secretion as a protective response [50]. Medium components can function as metabolic inducers; for example, ethanol or surfactants have been reported to stimulate EPS production in certain strains, whereas inhibitory compounds may suppress biosynthetic enzymes. Targeted nutrient pairing strategies, such as mannose combined with yeast extract in *Schizophyllum commune*, further illustrate how coordinated optimization can enhance output [53]. Collectively, EPS yield and structure emerge from the interaction of nutritional design, environmental control, and genetic regulation. Systematic integration of these variables is essential for improving productivity and achieving reproducible polymer specifications suitable for industrial deployment within circular bioeconomy frameworks.

## Downstream processing strategies determine fungal EPS quality and performance

The quality, structural integrity, and functional performance of fungal EPSs are strongly influenced by downstream processing strategies. While upstream parameters determine yield and intrinsic polymer architecture, extraction and purification workflows govern molecular preservation, impurity removal, and reproducibility across applications. Fungal EPSs produced by Ascomycota and Basidiomycota exhibit diverse physicochemical properties that directly affect recovery efficiency. Most are highly hydrophilic and readily soluble in water or physiological saline, whereas certain polymers require mild alkaline conditions (e.g., 1 M NaOH) for solubilization due to extensive inter-chain hydrogen bonding or rigid conformations [4]. Such solubility behavior influences extraction design, purification efficiency, and compatibility with biomedical or industrial formulations.

Although chemical composition and linkage diversity were discussed previously, these attributes become operationally relevant during downstream processing. Variations in monosaccharide composition, branching pattern, and substitution (e.g., acetylation or phosphorylation) affect precipitation behavior, chromatographic separation, and susceptibility to degradation [4, 63]. Molecular weight distribution, which may range from tens of kilodaltons to several megadaltons (Table 2), further determines viscosity, gelation potential, and biological diffusion properties. Reliable assessment of these parameters requires integrated analytical platforms, including chromatographic techniques (TLC, HPLC, GPC, IEC), spectroscopic analysis (FTIR, multi-dimensional NMR), and GC–MS profiling of monosaccharide residues [6]. These tools establish structure–activity relationships and support quality-control benchmarking.

**Table 2:** Comparative extraction, purification, and structural features of fungal exopolysaccharides

EPS producer	Extraction		Purification		Characterization		
	Precipitation method	Dialysis period (h)	EPS solution	Chromatographic methods	Composition	Linkage type	M_w_ (kDa)
*Aspergillus* sp. Y16	95% ethanol (1/3)	48	Water	IEC GPC	EPS1-Man, Gal EPS2-Man, Glc	EPS1—1 → 2;1 → 6, EPS2—1 → 3; 1 → 6	EPS1-15.0 EPS2-6.0
*Aspergillus versicolor*	95% ethanol (1/3)	48	Water	IEC GPC	Glc, Man	α-1 → 6	500.0
*Botryosphaeria rhodina* MMGR	Absolute ethanol (1/3)	48		GPC	Glc	β-1 → 6	-
*Penicillium commune*	95% ethanol (1/3)	48	Water	IEC GPC	Glc, Man, Gal	α-1 → 2 β-1 → 6	18.3
*Penicillium griseofulvum*	95% ethanol (1/3)	48	Water	IEC GPC	Man, Gal	α-1 → 2 β-1 → 5 α-1 → 6	-
*Fusarium oxysporum* JN604549	95% ethanol (1/3)	72	Water	IEC GPC	Gal, Glc, Man	β-1 → 6 β-1 → 2	61.2
*Fusarium oxysporum* Y24-2	95% ethanol (1/4)	72	Water	IEC GPC	Gal, Glc	α-1 → 2 β-1 → 6	36.0
*Fusarium solani* SD5	Absolute ethanol (1/5), 24 h, 4 °C	24	Water	GPC	Gal, Rha	α-1 → 2 β-1 → 4 α-1 → 6	187.0
*Grifola frondosa*	95% ethanol (1/4), 12 h, 4 °	–	–	GPC	–	–	EPS1-390,840.0 EPS2-3097.0
*Cryptococcus neoformans* ATCC 24,067	95% ethanol (1/3), CTAB-PS	24	Water	–	Xyl, GlcA, Man, Gal, Glc	–	1210.0
*Ganoderma lucidum* UF20706	95% ethanol (1/4), 12 h, 4 °C	–	–	HPAEC	EPS1-Rha, Glc EPS2-Rha, Gal	-	-
*Phellinus gilvus*	95% ethanol (1/4), 12 h, 4 °C	–	Buffer, pH 6.8	SEC/MALS	Mal, Arab, Xyl, Man, Gal, Glc	–	EPS1–8 628.0 EPS2–1 045.0 EPS3–610.9 EPS4–335.5
*Pleurotus sajor*-*caju*	95% ethanol	-	–	HPAEC	Man, Gal 3-*O*-methyl-Gal	1 → 6	64.0

*Arab* arabinose, *Fuc* fucose, *Gal* galactose, *Glc* glucose, *GlcA* glucuronic acid, *Mal* maltose, *Man* mannose, *Rha* rhamnose, *Xyl* xylose, *GPC* gel permeation chromatography, *SEC* size exclusion chromatography, *HPAEC* high performance anion exchange chromatography, *IEC* ion exchange chromatography, *SEC/MALS* the combination of SEC with multi-angle light scattering analysis (MALS). (Adopted from [63])

**Fig. 3:**
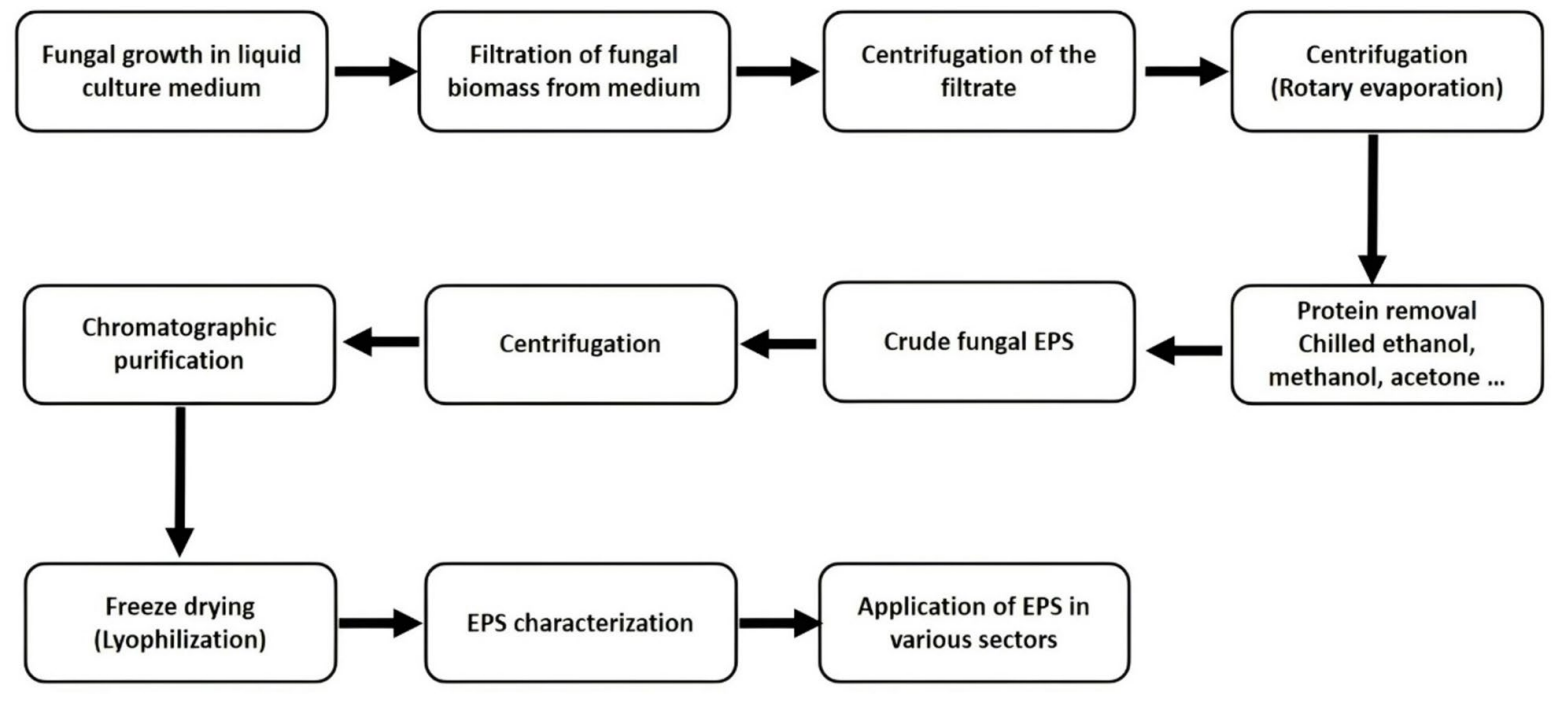
Workflow for extraction, purification, and downstream utilization of fungal exopolysaccharides (EPSs). Schematic overview of the downstream processing pipeline for fungal EPS production from submerged cultures. Following fungal growth in liquid medium, the biomass is separated from the culture broth by filtration. The clarified filtrate is then subjected to centrifugation and concentration steps, followed by precipitation and protein removal using organic solvents (e.g., chilled ethanol, methanol, or acetone) to obtain crude EPS. The crude polysaccharide is subsequently recovered by centrifugation and further purified through chromatographic techniques. Final processing includes freeze-drying (lyophilization), structural and physicochemical characterization, and deployment of purified EPS in various industrial, biomedical, environmental, and food-related applications

EPS recovery strategies depend on polymer localization and fermentation mode (Fig. 3). In submerged fermentation systems, extracellular EPSs can often be separated directly from culture supernatants via centrifugation or filtration. In contrast, capsular or intracellular fractions require mechanical or enzymatic cell disruption—such as homogenization, sonication, or cell wall digestion—prior to solubilization [4]. Regardless of source, alcohol precipitation remains the principal primary recovery method. Cold ethanol (typically 95%, v/v), methanol, acetone, or isopropanol is added at defined ratios—commonly 1:4 supernatant to solvent—and incubated at 4 °C for 12–24 h to induce polymer aggregation [27, 64, 65]. The resulting precipitate is collected by centrifugation and designated as crude EPS.

Crude preparations frequently contain co-precipitated proteins, pigments, lipids, and low-molecular-weight metabolites. Deproteinization is commonly achieved using Sevag reagent (chloroform: n-butanol, 4:1), which denatures and separates protein contaminants upon centrifugation [66]. Alternatively, trichloroacetic acid (5–13%) may be applied for selective protein precipitation [67]. Subsequent dialysis against deionized water (24–72 h) removes salts and small impurities. High-resolution purification typically employs chromatographic fractionation—ion-exchange chromatography (IEC), gel-permeation chromatography (GPC), size-exclusion chromatography with multi-angle light scattering (SEC-MALS), or high-performance anion-exchange chromatography (HPAEC)—to separate polymers based on charge density, hydrodynamic radius, or structural configuration [4]. These techniques enable isolation of defined molecular fractions suitable for functional testing or application-specific formulation.

For EPSs derived from fruiting bodies or immobilized biomass, additional extraction intensification methods may be required. Homogenization, microwave-assisted extraction, ultrasonic treatment, hot-water refluxing, subcritical water extraction, and enzyme-assisted hydrolysis have been employed to improve polymer release from complex matrices [2]. These approaches can enhance extraction kinetics and reduce solvent consumption, though careful control is required to avoid structural degradation of sensitive glycosidic linkages.

Despite methodological progress, harmonization of downstream workflows remains limited. Variability in solvent concentration, precipitation ratios, incubation time, pH adjustment, and deproteinization protocols can significantly alter molecular weight distribution and functional group integrity, complicating inter-study comparability [63]. Establishing standardized recovery and characterization panels is therefore essential for reproducibility, regulatory alignment, and scalable production. Integration of membrane filtration systems, automated chromatography, and solvent-reduced extraction technologies represents a logical progression toward industrial-scale refinement. In summary, downstream processing is not merely a recovery step but a defining determinant of fungal EPS performance. Careful control of extraction, purification, and analytical validation is required to translate biosynthetically diverse polymers into reproducible, application-ready biomaterials.

## Analytical challenges and strategies for accurate fungal EPS quantification

Accurate quantification of fungal EPSs is essential for evaluating fermentation performance, comparing production systems, and correlating polymer concentration with biological activity. However, reliable measurement is complicated by structural heterogeneity, variable monosaccharide composition, and co-extracted impurities. No single method provides universally accurate quantification; therefore, complementary analytical approaches are often required. The phenol–sulfuric acid assay remains the most commonly applied method for estimating total carbohydrate concentration due to its simplicity and compatibility with high-throughput screening. In this assay, acid hydrolysis releases monosaccharides that react with phenol and sulfuric acid to produce a chromophore measurable at 490 nm [68]. Although operationally robust, the assay reports total carbohydrate content rather than intact polymer mass. It may underestimate uronic acid–rich EPSs or overestimate yields when interfering substances are present. Quantitative accuracy is further influenced by the choice of calibration standard; glucose-based calibration can introduce bias when analyzing EPSs enriched in mannose, galactose, or other sugars. Matching standards to expected monosaccharide composition improves fidelity.

Gravimetric determination, based on drying and weighing alcohol-precipitated material, is also widely used. While straightforward, this method frequently overestimates true polysaccharide content because salts, pigments, lipids, and residual proteins co-precipitate during recovery. For this reason, gravimetric values should be interpreted cautiously and validated through carbohydrate-specific assays or purity assessment. Chromatographic techniques provide greater specificity. Gas chromatography (GC), high-performance liquid chromatography (HPLC), and GC–MS enable quantification of individual monosaccharides following hydrolysis and derivatization. GC–MS is particularly useful for mannose-rich polymers, where conversion to volatile derivatives (e.g., alditol acetates) permits sensitive mass detection [68]. However, derivatization steps increase analytical complexity and may introduce sample loss or variability. Detector sensitivity, hydrolysis efficiency, and incomplete derivatization can further influence reported yields, limiting routine applicability for large-scale process monitoring.

Additional sources of analytical uncertainty arise from heterogeneous molecular weight distributions, branching complexity, and the presence of protein–polysaccharide conjugates or phenolic contaminants that interfere with colorimetric and spectroscopic methods [4]. Consequently, discrepancies between reported yields across studies may reflect methodological differences as much as biological variation. As illustrated in Table 3, reported EPS yields span a broad range depending on fungal strain, cultivation conditions, and analytical approach. For example, *Botryosphaeria rhodina* DABAC-P82 produced 17.7 g/L under optimized conditions [69], and *Schizophyllum commune* LF01962 reached 18.28 g/L [53], whereas pharmaceutically investigated species such as *Antrodia camphorata* and *Ganoderma lucidum* often yield less than 1 g/L [31, 61]. These comparisons underscore the need for standardized quantification protocols when benchmarking productivity.

Improving analytical consistency requires harmonized calibration standards, combined gravimetric and carbohydrate-specific measurements, and transparent reporting of hydrolysis and derivatization conditions. Without methodological alignment, yield comparisons across laboratories remain difficult to interpret. Robust quantification frameworks are therefore central to process optimization, techno-economic evaluation, and regulatory acceptance of fungal EPS production systems.

**Table 3:** Representative high-yield fungal EPS production reports with cultivation context

Fungal species/strain	EPS yield (g/L)	Carbon source	Cultivation mode	Process status	Reference
*Botryosphaeria rhodina* DABAC-P82	17.7	Glucose	Submerged batch	Optimized	Selbmann et al., [69]
*Schizophyllum commune* LF01962	6.78	Glucose	Submerged batch	Baseline	Prathumpai et al., [53]
*Schizophyllum commune* LF01962	18.28	Glucose	Submerged batch	Optimized	Prathumpai et al., [53]
*Phoma herbarum* CCFEE 5080	13.6	Glucose	Submerged batch	Optimized	Selbmann et al., [70]
*Phellinus* sp. P0988	10.9	Glucose	Submerged batch	Optimized	Ma et al., [28]
*Morchella crassipes*	9.67	Carbohydrate medium	Submerged culture	Optimized	He et al., [66]
*Fusarium equiseti* ANP2	7.34	Glucose	Submerged batch	Optimized	Patel et al., [71]
*Ganoderma applanatum* GA2024	6.12	Glucose	Submerged batch	Optimized	Kim et al., [72]
*Ganoderma lucidum* HAI 447	1.6	Glucose	Submerged batch	Baseline	Elisashvili et al., [9]
*Cryptococcus laurentii* DSMZ 70,766	4.3	Glucose	Submerged aerobic	Optimized	Smirnou et al., [73]

## Advanced analytical approaches reveal fungal EPS structure–function relationships

Understanding structure–function relationships in fungal EPSs requires detailed molecular characterization beyond bulk carbohydrate quantification. EPSs are macromolecular assemblies composed of repeating monosaccharide units that may associate with extracellular DNA, glycoproteins, glycolipids, metal ions, and pigments within the extracellular matrix [4]. These interactions generate hydrated three-dimensional networks that influence adhesion, mechanical stability, and biological recognition. Fungal EPS diversity arises from variation in monosaccharide composition, glycosidic linkage patterns, branching frequency, stereochemistry, and substitution chemistry. Many EPSs are heteropolysaccharides composed primarily of hexoses such as glucose, mannose, and galactose, together with pentoses including xylose and arabinose. Additional components such as fucose, rhamnose, uronic acids, or sugar alcohols contribute to charge distribution and functional heterogeneity [67]. In contrast, certain genera produce homopolysaccharides composed exclusively of glucose residues. Linkage configuration strongly influences polymer conformation: β-(1→3) and β-(1→4) backbones are associated with increased rigidity and receptor recognition, whereas α-(1→2) and α-(1→6) linkages introduce flexibility that supports gel formation and emulsification (Table 2). Branched β-D-glucans with 1→6 side chains are widely reported among Ascomycota and Basidiomycota taxa [4].

Comprehensive structural elucidation relies on integrated analytical platforms. Monosaccharide composition is typically determined using HPLC or GC–MS following controlled hydrolysis and derivatization, enabling strain-specific compositional profiling. Functional group analysis by FTIR detects substitutions such as acetylation, phosphorylation, and sulfation, modifications that alter solubility, charge density, and receptor interaction. Nuclear magnetic resonance (¹H and ¹³C NMR) provides detailed information on anomeric configuration, linkage position, and repeating-unit architecture, supporting precise structural assignment. Chromatographic fractionation methods—including ion-exchange chromatography and size-exclusion chromatography (SEC/GPC/MALS)—define molecular weight distribution and branching characteristics, parameters directly linked to viscosity, gelation behavior, and immunological activity [64]. Emerging high-resolution techniques further expand structural insight. MALDI-TOF mass spectrometry enables oligomer profiling and detection of repeating units, while atomic force microscopy (AFM) and X-ray diffraction (XRD) provide information on supramolecular organization and crystallinity. These approaches help clarify how molecular arrangement influences mechanical stability, biofilm architecture, and host interaction.

Accurate structure–function mapping requires integrating compositional, conformational, and molecular-weight data. Without such multidimensional characterization, comparisons among EPSs from different fungal sources may be misleading, particularly when polymers share similar carbohydrate content but differ in linkage pattern or substitution chemistry. Rigorous structural analysis therefore underpins rational selection and design of fungal EPSs for defined biomedical, food, environmental, and materials applications.

## Translational deployment of fungal EPS across industrial sectors

The growing industrial relevance of fungal exopolysaccharides reflects increasing global demand for biologically derived polymers that combine functional performance with environmental compatibility. Unlike petrochemical materials, fungal EPSs offer structural tunability, biodegradability, and compatibility with sustainable manufacturing platforms. Commercially established polymers such as pullulan, schizophyllan, lentinan, scleroglucan, and grifolan already demonstrate validated biomedical, rheological, and barrier-forming performance. At the same time, emerging EPSs from unconventional and extremophilic fungi are expanding translational pipelines across healthcare, food, environmental biotechnology, and circular manufacturing systems.

### Fungal EPSs enable immunomodulatory and therapeutic biomedical applications

Fungal EPSs have been extensively investigated for bioactivity in cell-based and animal models, particularly for their capacity to influence immune signaling pathways. Most mechanistic evidence derives from in vitro macrophage and dendritic-cell systems or murine tumor models, where β-glucan–rich architectures interact with innate immune receptors such as dectin-1 (*CLEC7A*), *TLR2*, and complement receptor-3 (*ITGAM*) [4, 74]. Engagement of these receptors activates downstream signaling cascades involving SYK, CARD9, and MYD88, culminating in transcriptional activation of *NFKB1* and related immune regulators. Although these molecular interactions are well characterized experimentally, translation into standardized clinical protocols remains limited and highly context-dependent.

Among biomedical domains, oncology represents one of the most intensively studied areas. Polymers such as lentinan, schizophyllan, and grifolan have demonstrated immunostimulatory activity in preclinical systems. In these experimental models, enhanced macrophage activation and dendritic-cell maturation are associated with increased expression of cytokine genes including *IL6*, *IL12A*, *TNF*, and *IFNG*, which collectively stimulate cytotoxic T-cell and natural killer cell responses [74, 75]. Tumor suppression observed in these systems has also been correlated with downregulation of angiogenic mediators such as *VEGFA* and modulation of apoptosis-related regulators including *BAX* and *CASP3*. It is important to emphasize that the majority of these findings derive from controlled in vitro assays or animal tumor models.

Clinically, lentinan has been employed as an immunotherapeutic adjunct in oncology care, particularly in East Asia, where it is administered alongside conventional chemotherapy. In this setting, its role is generally interpreted as enhancement of host immune responsiveness rather than direct cytotoxic action [75]. Schizophyllan has likewise undergone clinical evaluation as an immune-supportive polysaccharide [63]. However, large-scale, multi-center randomized clinical trials outside specific regional approvals remain comparatively limited. Consequently, while gene-level immunomodulatory mechanisms are experimentally robust, broader oncological validation requires further rigorous clinical investigation.

Antiviral activity constitutes another area of investigation. In cell culture and small-animal infection models, EPS-mediated engagement of toll-like receptors activates interferon-associated pathways, inducing transcription of type I interferon genes *IFNA* and *IFNB1*, as well as interferon-stimulated genes such as *ISG15* and *MX1* [74, 76]. Reported reductions in influenza A replication, suppression of hepatitis B antigen release, and decreased herpes simplex virus infectivity [77–79] appear to involve both direct polymer–virus interactions and host immune modulation. Nevertheless, most antiviral findings remain preclinical, and controlled human trials are scarce. At present, antiviral positioning of fungal EPSs should therefore be considered investigational rather than clinically established.

Fungal EPSs have also demonstrated antioxidant and anti-inflammatory effects in experimental systems. These effects include modulation of inflammatory mediators such as *PTGS2* (COX-2), *TNF*, and *IL1B*, alongside suppression of signaling pathways mediated by *NFKB1* and mitogen-activated protein kinases including *MAPK1* and *MAPK8* [4, 74]. Concurrent upregulation of antioxidant enzyme genes, including *SOD1* and *CAT*, has been associated with improved cellular resistance to oxidative stress. While these molecular effects are reproducible in vitro and in animal models, clinical dose–response relationships, pharmacokinetics, and long-term safety parameters remain insufficiently characterized for most EPS systems.

In regenerative medicine, fungal EPSs are investigated as components of wound dressings and tissue-engineering scaffolds. EPS-based hydrogels have been shown in experimental models to promote expression of genes involved in tissue remodeling and angiogenesis, including *TGFB1*, *VEGFA*, and collagen-associated genes such as *COL1A1* [75]. Clinical observations report improved wound outcomes using β-glucan–based dressings, while pullulan-based scaffolds have demonstrated osteogenic support and enhanced tissue stability in vivo [80–82]. However, many regenerative applications remain supported primarily by small cohort studies or experimental implantation models.

Beyond direct therapeutic use, EPS chemical versatility enables development of controlled delivery systems. Pullulan derivatives have been investigated for mucosal drug transport and hepatocyte-targeted nucleic acid delivery in preclinical models [83, 84]. Nano-engineered EPS platforms are also being explored as non-viral carriers in gene delivery strategies [24], though translation to clinical gene therapy remains at an early developmental stage. Fungal EPSs further exhibit potential as vaccine adjuvants by enhancing antigen uptake, dendritic-cell activation, and systemic and mucosal antibody responses. Experimental studies report improved immune memory and T-cell activation using lentinan-based composite formulations [66, 76]. At present, most data derive from animal immunization models, and standardized clinical adjuvant approval pathways have yet to be broadly established.

Collectively, fungal EPSs demonstrate substantial immunological and biomaterial functionality across multiple biomedical domains. However, the level of evidence varies considerably between applications, ranging from mechanistic in vitro studies and animal models to regionally approved adjunct therapies. Clear differentiation between experimental immunomodulation and globally validated clinical efficacy is essential for responsible translational positioning. Representative EPS types, production features, and biomedical applications are summarized in Table 4.

**Table 4:** Integrated techno-biological profile of major fungal EPSs linking cultivation parameters, structural characteristics, and translational status

Source organism	EPS	Production platform and key parameters	Substrate used	Yield range	Applications	Evidence maturity/market status	References
*Aureobasidium pullulans*	Pullulan	Submerged aerobic fermentation; 25–30 °C; pH 5–6; controlled aeration	Glucose, sucrose, starch hydrolysates	20–35 g/L	Pharmaceutical capsules; edible films; drug delivery matrices	Commercially established in food and pharma excipients	Sugumaran and Ponnusami, [34]; Singh et al., [85]
*Schizophyllum commune*	Schizophyllan	Controlled submerged fermentation; pH 4–5; high aeration	Glucose; agro-residues	15–18 g/L	Oncology adjunct; immune modulation	Regional clinical use (Japan); strong preclinical support	Zhang et al., [86]; Kumar et al., [35]
*Sclerotium rolfsii*	Scleroglucan	Industrial aerobic fermentation; 28–30 °C; optimized agitation	Glucose; starch mixtures	20–30 g/L	Rheology modifier; enhanced oil recovery; drug delivery research	Commercial in industrial formulations	Survase et al., [36]; Jindal and Khattar (2018)
*Botryosphaeria rhodina*	Botryosphaeran	Submerged fermentation; optimized C: N ratio	Glucose; sucrose	15–18 g/L	Anticoagulant derivatives; antioxidant research	Experimental (in vitro + animal models)	Mendes et al., [87]; Geraldelli et al., [37]
*Lentinula edodes*	Lentinan	Fruiting body extraction; hot-water extraction	Solid-state cultivation on lignocellulosic substrates	Extract-based (1–5% dry wt)	Cancer immunotherapy adjunct	Regionally approved adjunct therapy	Chakraborty et al., [88]; Sobieralski et al., [38]
*Lasiodiplodia theobromae*	Lasiodiplodan	Submerged fermentation on agro-residues; ~28 °C	Molasses; agro-industrial wastes	10–20 g/L	Antioxidant and biomaterial derivatives	Preclinical stage	Kagimura et al., [89]; Abdeshahian et al., [90]
*Grifola frondosa*	Grifolan	Biomass extraction from cultivated fruiting bodies	Mushroom substrate	Extract-based	Immunopharmacological and nutraceutical applications	Nutraceutical markets; preclinical support	Ishibashi et al., [91]; Chakraborty et al., [92]

### Skin-active fungal EPSs drive innovation in cosmeceutical formulations

Fungal EPSs are increasingly incorporated into dermatological and cosmetic formulations due to their film-forming capacity, hydration retention, and formulation stability. Their hydrophilic polymer networks interact strongly with water molecules, creating semi-occlusive surface films that enhance skin viscoelasticity and reduce transepidermal water loss [4, 12]. Beyond surface hydration, emerging in vitro and ex vivo evidence suggests that certain fungal EPSs may influence epidermal barrier function through modulation of genes associated with structural integrity, including *FLG* (filaggrin), *LOR* (loricrin), and *IVL* (involucrin), which are essential for stratum corneum maturation. Increased expression of tight junction components such as *CLDN1* and *OCLN*, together with upregulation of aquaporin-3 (*AQP3*), has been reported in keratinocyte models, suggesting potential reinforcement of barrier cohesion and water transport. While these findings support a mechanistic basis for barrier enhancement, clinical confirmation of long-term gene-level effects in human skin remains limited.

Polysaccharides from *Tremella fuciformis* have received particular attention because of their hygroscopic behavior, often compared to hyaluronic acid in terms of surface hydration performance. Controlled clinical evaluations demonstrate improved stratum corneum hydration without irritation, supporting their application as natural humectants in sensitive-skin formulations [93]. However, these outcomes primarily reflect short-term hydration metrics rather than gene-level remodeling. Similarly, EPS-enriched extracts from *Volvariella volvacea* have been associated in experimental models with modulation of melanogenesis-related genes such as *MITF*, *TYR*, and *TYRP1*, alongside increased expression of extracellular matrix–associated genes including *COL1A1*, *COL3A1*, and elastin (*ELN*) [94]. These transcriptional changes were observed predominantly in cell-based systems and are interpreted as indicative of potential dermal-supportive effects. Although improvements in firmness and pigmentation uniformity have been reported in small-scale cosmetic evaluations, large-scale randomized dermatological trials remain limited.

In addition to hydration and matrix-related responses, fungal EPSs contribute to photoprotection and barrier support through antioxidant mechanisms. Experimental studies indicate activation of cytoprotective pathways regulated by nuclear factor erythroid 2–related factor 2 (*NFE2L2*), resulting in increased transcription of downstream antioxidant genes such as *HMOX1*. Concurrent suppression of matrix-degrading enzymes including *MMP1* and *MMP3* has been observed in UV-exposed keratinocyte and fibroblast models, suggesting potential reduction of collagen degradation and photoaging-related processes. These findings are primarily derived from in vitro systems and provide mechanistic insight rather than direct clinical proof of anti-aging efficacy. β-Glucan–containing formulations derived from *Pleurotus ostreatus* have demonstrated clinical benefit in individuals with atopic-prone skin, including improvement in symptom severity and barrier recovery following topical application [95]. These effects are consistent with experimental observations of enhanced keratinocyte proliferation markers such as *KRT14* and normalization of differentiation-associated genes including *KRT10*. Nevertheless, broader dermatological validation across diverse populations and long-term usage conditions remains necessary.

Beyond biological activity, fungal EPSs provide technological advantages in formulation science. Polymers from *Cordyceps militaris* improve emulsion stability, viscosity control, and long-term shelf performance while maintaining desirable sensory characteristics [96]. Their multifunctionality as rheology modifiers and stabilizers reduces reliance on synthetic additives in clean-label cosmetic systems [75]. EPSs produced by *Rhodotorula mucilaginosa* have also demonstrated supportive roles in post-procedure skin recovery in experimental models through promotion of keratinocyte proliferation and dermal remodeling [12].

Collectively, fungal EPSs exhibit hydration-enhancing, barrier-supportive, antioxidant, and matrix-modulating properties at both physicochemical and transcriptional levels. However, much of the mechanistic evidence derives from in vitro and small-scale clinical studies. Future progress in this domain will depend on well-designed randomized dermatological trials, standardized gene-expression profiling in human skin, and long-term safety assessment to distinguish cosmetic functionality from clinically validated dermatotherapy.

### Fungal EPSs support functional performance in industrial bioprocesses

Fungal EPSs function as versatile biomacromolecules in industrial processing systems due to their tunable rheology, high molecular weight, branching architecture, and surface-binding capacity. These physicochemical properties enable viscosity modulation, emulsification, film formation, and stabilization across environmental, food, and materials-engineering platforms [4, 12]. In water and wastewater treatment, fungal EPSs serve as biodegradable bioflocculants that aggregate suspended solids, emulsified oils, and dissolved metal ions through electrostatic interactions and chelation mechanisms [12]. Compared to synthetic flocculants, EPS-based clarification improves sludge dewaterability while reducing residual toxicity, supporting their application in environmentally regulated treatment facilities [20, 97].

In materials engineering and packaging industries, fungal EPSs contribute to the development of biodegradable films, coatings, and nanostructured materials with desirable oxygen and moisture barrier properties [98]. Pullulan demonstrates high transparency and gas impermeability due to its α-(1→6)/(1→4) glycosidic structure, supporting its use in pharmaceutical capsules and edible films [85, 99]. Scleroglucan maintains viscosity under high salinity, thermal stress, and mechanical shear, enabling its use in industrial formulations requiring structural stability [97].

Within food systems, fungal EPSs function primarily as natural rheology modifiers that enhance viscosity, texture, and product stability without adversely affecting flavor profiles [4]. Pullulan has been widely applied in confectionery coatings and bakery glazes due to its neutral sensory characteristics and film-forming ability [85, 100]. Scleroglucan provides processing stability during freezing–thawing cycles and thermal sterilization, expanding its use in frozen and heat-treated foods [97]. Lentinan- and pleuran-enriched formulations have also been incorporated into functional dairy matrices to improve product consistency and nutritional positioning [101].

Fungal EPSs additionally contribute to the nutraceutical sector as fermentable polysaccharides capable of modulating gut microbiota composition. Extracts from *Ganoderma lucidum*, *Aureobasidium pullulans*, *Pleurotus* spp., and *Lentinula edodes* have been associated with stimulation of beneficial bacterial populations such as *Bifidobacterium* and *Lactobacillus* [102]. These applications emphasize metabolic and microbiome-supportive functions rather than direct pharmacological effects, distinguishing industrial food use from the therapeutic contexts described above. Overall, the industrial utility of fungal EPSs derives from their structural adaptability, rheological performance, and compatibility with food-grade and environmentally regulated processing systems. Continued improvements in fermentation scalability and downstream refinement are expected to further expand their integration into materials engineering, environmental treatment, and food biotechnology platforms [20, 98].

### Environmental and agricultural applications exploit the multifunctionality of fungal EPS

Fungal EPSs play important roles in environmental engineering systems where structural stability, adsorption capacity, and tolerance to extreme conditions are required. In enhanced oil recovery (EOR), fungal EPSs such as schizophyllan and scleroglucan exhibit high viscosity retention under elevated salinity, temperature, and pressure, enabling improved displacement of residual hydrocarbons within porous rock formations [103]. Schizophyllan has been reported to enhance crude oil mobilization beyond residual saturation levels, demonstrating competitive performance relative to conventional polymeric injectants [104]. Chemical modification strategies, including hydrophobic derivatization and sulfonation of scleroglucan, further improve reservoir compatibility and viscosity stability in brine environments [105]. These adaptations highlight the suitability of fungal EPSs for subsurface resource-engineering applications.

In environmental remediation, fungal EPSs contribute to pollutant immobilization through adsorption and complexation mechanisms. Functionalized polysaccharide chains bind heavy metals and organic contaminants via chelation and ion exchange, reducing their mobility in soils and aqueous systems [12, 63]. Uronic acid–rich heteropolysaccharides demonstrate strong affinity for metal ions such as Pb²⁺, Cd²⁺, and Cu²⁺, supporting their application in stabilization of contaminated environments. These properties enable in situ remediation approaches that rely on biological sequestration rather than synthetic chemical agents. In agricultural soils, fungal EPS secretion contributes to structural stabilization of the rhizosphere. Extracellular polysaccharides enhance soil particle aggregation, increase porosity, and improve water retention capacity, thereby supporting plant growth under stress-prone conditions [20, 106]. By reinforcing microhabitat formation around roots, EPSs promote beneficial microbial colonization and nutrient exchange dynamics. Experimental studies demonstrate that EPS-based seed coatings can improve germination rates and reduce incidence of soil-borne phytopathogens [107]. Additional investigations report enhanced root elongation and seedling biomass in horticultural crops following application of EPS-producing fungal strains [107].

Beyond crop systems, fungal EPSs are increasingly explored in livestock and aquaculture nutrition as feed additives that support gut integrity and performance stability. Supplementation with Ganoderma-derived EPS has been associated with improved lipid profiles and modulation of gut microbial composition in poultry systems [108]. β-Glucan supplementation in fish and swine production has been linked to improved survival and growth performance under microbial challenge conditions [109, 110]. These applications illustrate how fungal polysaccharides contribute to reduced antibiotic reliance and improved resilience in animal production systems. Collectively, environmental and agricultural deployments of fungal EPSs demonstrate their utility in subsurface engineering, pollutant immobilization, soil stabilization, and sustainable livestock management. These domains emphasize functional performance within ecological and resource-management contexts, complementing the biomedical and industrial applications discussed in preceding sections.

### Industrial production and circular bioeconomy integration of fungal EPS

Industrial production of fungal exopolysaccharides has advanced from laboratory-scale cultivation to specialized commercial biopolymer manufacturing, particularly for β-glucans and film-forming polymers such as pullulan, schizophyllan, lentinan, and scleroglucan [75]. Current production systems predominantly rely on submerged aerobic fermentation in stirred-tank bioreactors operated under controlled temperature (22–30 °C), pH (3.0–6.5), and aeration conditions, where oxygen transfer rates and shear dynamics directly influence molecular weight distribution and overall productivity [2]. At this scale, process reproducibility becomes as critical as strain selection. Economic feasibility is closely linked to substrate strategy. Replacement of refined sugars with agro-industrial side streams—including molasses, starch residues, lignocellulosic hydrolysates, and brewer’s spent grain—reduces feedstock cost while maintaining competitive yields [111]. For example, fermentation of *Schizophyllum commune* using brewer’s spent grain as the sole carbon source achieved production levels comparable to refined media [15]. Such integration into biorefinery workflows demonstrates that EPS production can operate within waste-valorization systems without compromising performance.

Commercial trajectories illustrate how functional value drives industrial adoption. Pullulan remains one of the most mature fungal EPS products due to its superior film-forming and oxygen-barrier properties, supporting pharmaceutical capsules and edible coatings [75]. Schizophyllan and lentinan occupy higher-value biomedical niches, whereas scleroglucan has achieved broader industrial deployment because of its stability under elevated temperature and salinity [112]. In each case, market success reflects alignment between intrinsic polymer architecture and application-specific performance requirements rather than mere production capacity.

A central challenge in scaling fungal EPS systems lies in reconciling structural diversity with manufacturing standardization. Fungal strains inherently synthesize polymers with distinct monosaccharide compositions, branching frequencies, and molecular weights. However, industrial standardization does not necessitate structural uniformity; it requires process harmonization. Fermentation platforms can be standardized through controlled bioreactor operation, oxygen-transfer regulation, defined substrate regimes, and statistical optimization models, while strain identity defines the final polymer architecture. In this framework, strain diversity becomes a design variable rather than a manufacturing obstacle.

Downstream recovery similarly follows a conserved backbone workflow—biomass removal, alcohol precipitation, deproteinization, dialysis, and chromatographic refinement—that can be modularly adjusted according to purity specifications. Analytical harmonization further supports scalability through standardized quality-control panels including monosaccharide profiling, molecular weight distribution analysis (SEC/GPC), FTIR/NMR verification, and contaminant assessment [75]. When integrated into platform-based manufacturing, these workflows enable reproducible product specifications despite underlying biological variability. Patent activity and bibliometric trends indicate sustained expansion of fungal polysaccharide technologies across pharmaceutical, food, agricultural, and environmental sectors [2]. Nonetheless, widespread deployment remains constrained by strain-dependent productivity, purification cost, and regulatory harmonization requirements. Addressing these constraints requires integration of synthetic biology, continuous fermentation systems, membrane-assisted purification, and techno-economic modeling to improve consistency and cost efficiency.

Viewed through a systems perspective, fungal EPS industrialization is evolving toward adaptable manufacturing platforms embedded within biorefinery infrastructures. Standardized process control coupled with strain-specific design logic provides a scalable pathway for translating molecular diversity into reproducible, regulatory-aligned products. This shift from strain-centric production toward harmonized platform engineering represents a decisive step in enabling broad deployment of fungal EPSs across biomedical, material, and environmental technologies.

## Overcoming biosynthetic, processing, and regulatory barriers in fungal EPS commercialization

Despite substantial progress in fermentation engineering and application development, fungal EPS commercialization remains constrained by three interdependent bottleneck domains: biosynthetic control, downstream standardization, and regulatory harmonization. Biosynthetic constraints arise from incomplete mechanistic understanding of gene-cluster organization, enzyme specificity, and secretion dynamics. Strain-dependent variability in glycosyltransferase repertoires and sugar-nucleotide flux results in fluctuations in molecular weight, branching architecture, and substitution patterns. While such variability can enable functional diversity, it complicates reproducible manufacturing. Limited resolution of transcriptional regulation and pathway integration has also slowed adoption of predictive metabolic engineering strategies capable of delivering application-specific polymer architectures [27]. Addressing this constraint requires systematic integration of genomics, transcriptomics, and metabolomics to map pathway control points and enable targeted strain optimization.

Processing constraints primarily concern purification and scale-up reproducibility. EPSs frequently co-precipitate with proteins, pigments, and cell-wall fragments during recovery, introducing variability in purity and physicochemical characteristics that directly affect product standardization [63]. At industrial scale, additional challenges include oxygen-transfer limitations, foam formation, sterilization logistics, and shear-induced molecular degradation [20]. Substitution of refined substrates with lignocellulosic residues or agro-industrial side streams can reduce production cost but introduces compositional heterogeneity requiring adaptive process control [113, 114]. Progress therefore depends on intensified fermentation strategies, membrane-assisted separations, and automation-supported quality monitoring to ensure batch-to-batch consistency.

Regulatory constraints remain decisive for medical and food-grade deployment. High-value EPS applications demand standardized chromatographic and spectroscopic characterization, toxicological validation, and traceability systems aligned with international manufacturing compliance standards [115]. The absence of harmonized regulatory frameworks across jurisdictions prolongs approval timelines and discourages large-scale investment. Establishing universally accepted quality-control panels and specification benchmarks is therefore essential for accelerating market entry.

**Fig. 4:**
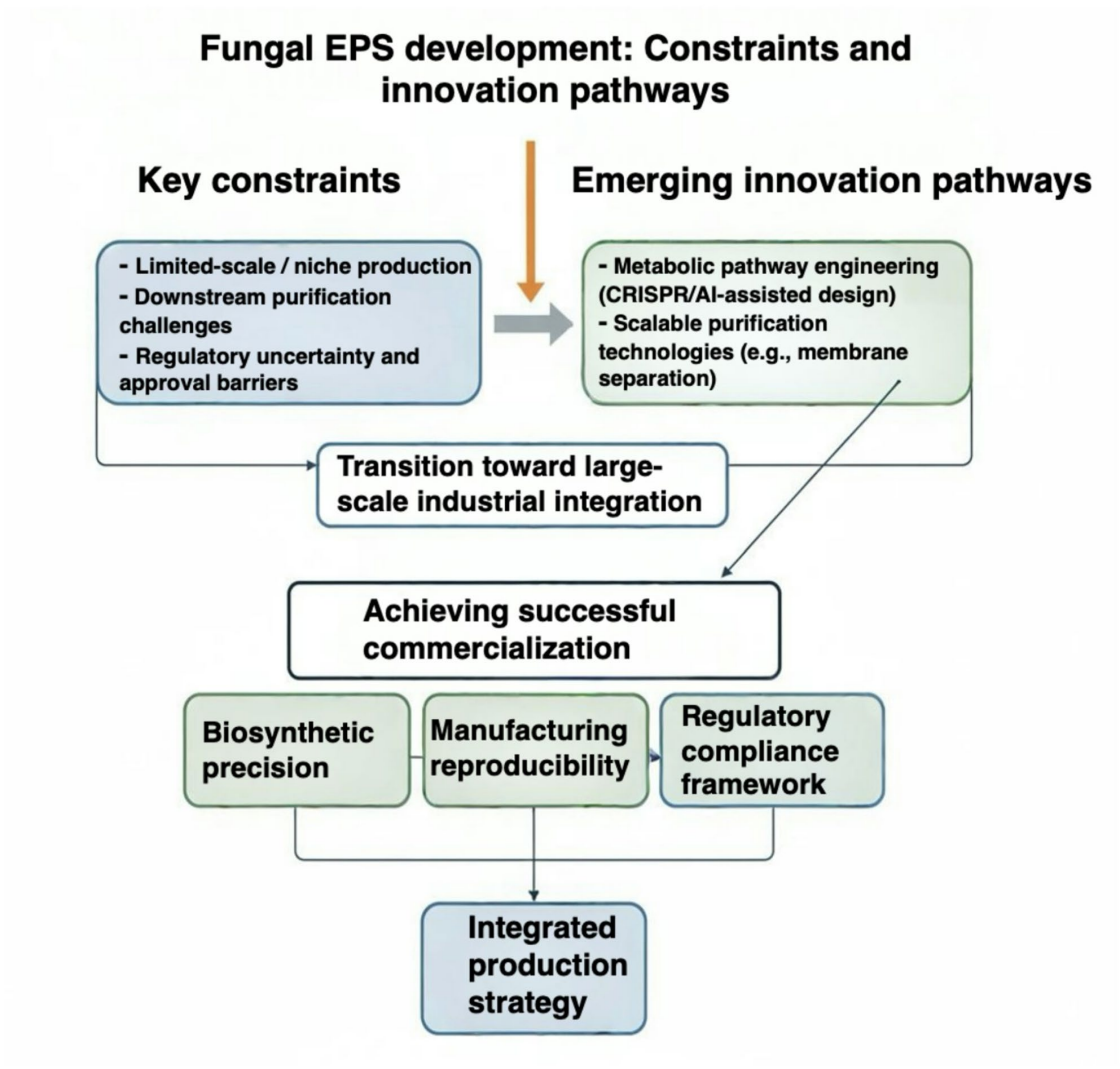
Conceptual framework for fungal exopolysaccharide (EPS) development. Key constraints (limited-scale production, purification challenges, and regulatory uncertainty) and innovation pathways (metabolic engineering, scalable purification, and standardization) enabling industrial integration and successful commercialization through biosynthetic precision, manufacturing reproducibility, and regulatory compliance

Technological developments are converging toward resolution of these bottlenecks. Multi-omics analytics and CRISPR-enabled pathway engineering are improving biosynthetic predictability, while machine learning approaches enhance structure–function modeling [116, 117]. Continuous fermentation, membrane-integrated bioprocessing, and digital process control systems are strengthening manufacturing robustness. Exploration of extremophilic fungi is expanding access to polymers with intrinsic stability under harsh environmental conditions, potentially reducing downstream modification requirements [118]. Figure 4 summarizes the primary constraints and projected innovation trajectories guiding fungal EPS development. Transition from niche production to broad industrial integration will depend on coordinated advances in pathway-level engineering, scalable purification technologies, and regulatory standardization. Within this framework, successful commercialization is not determined solely by polymer novelty, but by the ability to align biosynthetic precision, manufacturing reproducibility, and compliance architecture into a unified production strategy.

## Conclusion

Fungal EPSs have evolved from being regarded as secondary microbial metabolites to being understood as architecturally programmable polymer systems. The synthesis of ecological diversity, structural classification, gene-cluster regulation, process engineering, and industrial translation presented in this review demonstrates that EPS functionality emerges from coordinated multi-level control rather than isolated compositional traits. Polymer performance reflects the interaction of lineage-specific biosynthetic capacity, metabolic flux regulation, cultivation environment, molecular architecture, and downstream specification. A central conceptual shift highlighted in this work is the reinterpretation of biological variability. Structural heterogeneity among fungal strains, once considered a barrier to commercialization, can function as a tunable design dimension when embedded within harmonized fermentation platforms and standardized analytical workflows. Under such a framework, strain diversity expands the accessible material design space without compromising manufacturing reproducibility. Future advancement will depend on tighter integration between biosynthetic gene-cluster knowledge, predictive metabolic modeling, controlled bioprocess engineering, and robust quality-control systems. The convergence of synthetic biology, multi-omics analytics, continuous fermentation technologies, and advanced structure–function modeling will enable deliberate modulation of branching architecture, substitution patterns, and molecular weight distribution to meet defined application requirements. As process standardization and regulatory harmonization mature, fungal EPSs are positioned not merely as alternative biopolymers, but as adaptable material platforms capable of supporting biomedical innovation, resilient industrial systems, and sustainability-oriented manufacturing strategies.

## Data Availability

Data will be given upon request.
